# Molecular Evolution of the *Fusion* (*F*) Genes in Human Metapneumovirus Genotype B

**DOI:** 10.3390/microorganisms14020396

**Published:** 2026-02-06

**Authors:** Tatsuya Shirai, Fuminori Mizukoshi, Mitsuru Sada, Kazuya Shirato, Takeshi Saraya, Haruyuki Ishii, Ryusuke Kimura, Toshiyuki Sugai, Akihide Ryo, Hirokazu Kimura

**Affiliations:** 1 Department of Respiratory Viruses, National Institute of Infectious Diseases, Japan Institute for Health Security, Musashimurayama-shi 208-0011, Tokyo, Japan; shirai.t@jihs.go.jp (T.S.); shirato.k@jihs.go.jp (K.S.); 2 Department of Respiratory Medicine, Faculty of Medicine, Kyorin University, Mitaka-shi 181-8611, Tokyo, Japan; rainbow_orch@ks.kyorin-u.ac.jp (M.S.); saraya@ks.kyorin-u.ac.jp (T.S.); h141@ks.kyorin-u.ac.jp (H.I.); 3 Advanced Medical Science Research Center, Gunma Paz University, Takasaki-shi 370-0006, Gunma, Japan; 4 Department of Bioinformatics and Integrative Omics, National Institute of Infectious Diseases, Japan Institute for Health Security, Musashimurayama-shi 208-0011, Tokyo, Japan; mizukoshi.f@jihs.go.jp; 5 Gunma Prefectural Institute of Public Health and Environmental Sciences, Maebashi-shi, Gunma 371-0052, Gunma, Japan; kimura-r56@pref.gunma.lg.jp; 6 Department of Health and Welfare, Faculty of Health and Welfare, Prefectural University of Hiroshima, Mihara-shi 723-0053, Hiroshima, Japan; sugai@pu-hiroshima.ac.jp; 7 Department of Health Science, Graduate School of Health Sciences, Gunma Paz University, Takasaki-shi 370-0006, Gunma, Japan

**Keywords:** conformational epitopes, fusion gene, human metapneumovirus, molecular evolution

## Abstract

Human metapneumovirus genotype B (HMPV-B) is an important respiratory pathogen, requiring detailed elucidation of the evolutionary and antigenic features of its *fusion* (*F*) gene. Using 500 sequences collected between 1982 and 2024, we investigated the molecular evolution, phylodynamics, and structural epitope landscape of the HMPV-B *F* gene. Time-scaled phylogeny dated the divergence of sublineages B1 and B2 to around 1937, and Bayesian Skyline Plot analysis showed that these sublineages exhibited distinct demographic trajectories over time. The *F* gene evolved at a rate of 1.01 × 10^−3^ substitutions/site/year; however, amino acid variation remained limited, consistent with pervasive purifying selection, with 39% of codons under strong negative selection and little consensus evidence for positive selection. Conformational B-cell epitope prediction demonstrated a high degree of conservation across neutralizing antibody binding regions (sites Ø and I–V), and amino acid substitutions occurring within these sites were not predicted to substantially alter epitope architecture. Together, these findings indicate that the HMPV-B *F* gene evolves under strong evolutionary constraint while maintaining stable antigenic features, supporting the potential for antibody-based strategies that target neutralizing antibody binding regions of the F protein.

## 1. Introduction

Human metapneumovirus (HMPV), a pathogen of the family Pneumoviridae (genus *Metapneumovirus*) first identified in 2001, is a significant cause of acute respiratory infections (ARIs) globally [1]. Reflecting its widespread circulation, infection occurs predominantly in early childhood, with an estimated 50% of children becoming infected by age two and nearly all by age five [1]. The clinical manifestations of HMPV are diverse, ranging from mild upper respiratory illness to severe conditions such as bronchiolitis and pneumonia, which are estimated to be responsible for 6.2% of hospital admissions for ARI [2]. Moreover, the clinical burden of HMPV is amplified in the context of co-infections with other pathogens, such as bacteria and fungus, which can lead to critical and potentially lethal complications, especially in vulnerable populations [3,4]. This clinical challenge is compounded by the absence of licensed vaccines or specific antiviral therapies, leaving supportive care as the sole management strategy. Thus, HMPV infection represents a significant public health concern.

As an enveloped RNA virus, HMPV possesses a genome of approximately 13 kb containing eight genes. These genes encode nine proteins, three of which—the fusion (F), attachment (G), and small hydrophobic (SH) proteins—are surface glycoproteins crucial for viral function. Genetic diversity, particularly within the *F* and *G* genes, provides the basis for classifying HMPV into two genetic lineages, designated genotypes A (HMPV-A) and B (HMPV-B) [5,6,7]. These genotypes were traditionally further divided into the sublineages A1, A2, B1, and B2 [8]. More recently, a unified numeric nomenclature has been proposed to better reflect ongoing viral evolution [9]. Under this system, genotype B remains classified as B1 and B2, whereas genotype A is subdivided into A1, A2.1, and A2.2 (comprising A2.2.1 and A2.2.2) [9]. The prevalence of these genotypes and sublineages varies temporally and geographically, and understanding their circulation dynamics is crucial for public health surveillance. HMPV-B, in particular, co-circulates with other respiratory viruses or independently causes significant seasonal outbreaks [10,11]. Furthermore, during the increase in HMPV infections reported in China in late 2024, molecular epidemiologic investigations indicated that the majority of circulating strains belonged to the B2 sublineage of HMPV-B, supporting the epidemiologic significance of genotype B [12,13].

HMPV is phylogenetically and clinically related to human respiratory syncytial virus (RSV), another major pathogen within the Pneumoviridae family [14]. For both viruses, the F protein is the primary target of the host neutralizing antibody response, making it the central focus for vaccine and antibody-based intervention development. Extensive research on RSV has identified at least six antigenic sites on its F protein (designated sites Ø, I, II, III, IV, and V), which serve as a valuable framework for understanding pneumovirus antigenicity [15,16]. While sites Ø and V are the targets of the most potent neutralizing antibodies in RSV, the corresponding region in HMPV is shielded by a dense network of N-linked glycans, which limits its immunogenicity [17,18]. Sites I and II have also been identified as potential targets for neutralizing antibodies in HMPV, although reports characterizing them remain limited [19]. In contrast, the regions corresponding to sites III and IV in HMPV emerge as some of the most accessible and critical targets for neutralizing antibodies [19]. Furthermore, sites III and IV are structurally highly conserved between HMPV and RSV, identifying them as prime targets for cross-reactive antibodies capable of neutralizing both viruses [16].

The F protein is relatively conserved compared to the highly variable G protein, rendering it a promising target for antibody-based interventions and vaccine development. Nevertheless, amino acid substitutions can accumulate during viral circulation, potentially altering antigenicity. In addition, experience from RSV has demonstrated that even limited, subgroup-specific amino acid substitutions in the F protein, such as the emergence of two substitutions in RSV-B (L172Q/S173L), can abrogate monoclonal antibody neutralization and lead to clinical failure, underscoring the need for genotype-specific, high-resolution molecular analyses [20]. Despite the clinical importance of HMPV-B, comprehensive molecular evolutionary analyses of its *F* gene remain limited. To address this gap, the present study integrates phylogenetic and in silico approaches to elucidate the molecular evolution of the HMPV-B *F* gene, with the aim of characterizing its genetic diversity and predicting potential changes in antigenic sites.

## 2. Materials and Methods

### 2.1. Data Collection and Sequence Retrieval

We obtained the full-length coding region of the HMPV-B *F* gene (positions 3052–4671; 1620 nt for HMPV strain; NCBI Reference Sequence: NC_039199.1) from NCBI Virus (https://www.ncbi.nlm.nih.gov/labs/virus/vssi/#/ (accessed on 20 February 2025)) to analyze the molecular evolution. The initial dataset was curated by first excluding sequences that contained ambiguous nucleotides (e.g., N, Y, R, or V) or lacked clear collection dates or geographic origins. From the remaining sequences, only those belonging to genotype B were selected for further analysis, based on the genotyping scheme from Nextstrain (https://clades.nextstrain.org/ (accessed on 14 March 2025)) [21]. Then, duplicate sequences (100% identity) were identified using Clustal Omega (https://www.ebi.ac.uk/jdispatcher/msa/clustalo (accessed on 26 March 2025)) [22], and within each group of identical sequences, only the strain with the earliest sampling year was retained, while the others were excluded. Subsequently, five outlier sequences considered to be potential sequencing artifacts were removed to prevent bias: two (JQ413397.1, JQ413401.1) for containing large internal deletions, and three (MK177081.1, MK177095.1, MK177098.1) for exhibiting an unusually high number of unique substitutions. These outliers were considered potential artifacts, such as sequencing errors, which could bias the results of the molecular evolutionary analysis. This curation resulted in a dataset of 509 sequences. Because the Datamonkey web server used for selective pressure analysis limits the number of sequences to 500 per analysis [23], the dataset was randomly downsized to 500 sequences (B1: 212 sequences; B2: 288 sequences) without regard to sublineage or sampling year. Details of randomly excluded strain are shown in Supplementary Table S1. The final dataset comprised strains from 18 countries spanning 1982 to 2024 (Supplementary Figure S1), with detailed information provided in Supplementary Table S2.

### 2.2. Time-Scaled Phylogenetic Tree

A time-scaled phylogenetic tree of the HMPV-B *F* gene was constructed using the Bayesian Markov chain Monte Carlo (BMCMC) approach in BEAST version 2.6.7 [24]. For this analysis, the core dataset of 500 HMPV-B sequences was supplemented with a single HMPV-A reference strain (GenBank accession no. NC_039199.1) as an outgroup to estimate the divergence time between HMPV-A and HMPV-B; thus, the phylogenetic tree construction was performed on 501 sequences in total. Prior to the BMCMC analysis, the optimal nucleotide substitution model (TrN + I + G) was determined using jModelTest version 2.1.10 [25]. Subsequently, path-sampling and stepping-stone sampling methods were employed to compare four molecular clock models (strict, relaxed exponential, relaxed log normal, and random local) and two coalescent tree priors (constant population and exponential population). This comparison identified the relaxed exponential clock and the coalescent constant population model as the best fit for the data. The BMCMC analysis was run for 300 million steps, sampling every 3000 steps. Convergence of the chains was assessed in Tracer version 1.7.2 [26], and an effective sample size (ESS) greater than 200 was accepted for all parameters. The first 15% of samples were discarded as burn-in. The maximum clade credibility (MCC) tree was summarized using TreeAnnotator version 2.6.7 and visualized with FigTree version 1.4.0 (http://tree.bio.ed.ac.uk/software/figtree/). Branch support was evaluated using 95% highest posterior density (HPD) intervals. Detailed parameter settings used in the BMCMC analyses are provided in Supplementary Table S3.

### 2.3. Bayesian Skyline Analysis and Substitution Rate Estimation

We investigated the historical population dynamics of the HMPV-B *F* gene using a Bayesian skyline plot (BSP) model implemented in the BEAST package. The analysis, aimed at estimating changes in effective population size over time, was applied to both the full set of 500 HMPV-B sequences and to each sublineage (B1 and B2). The best substitution and clock models were selected, as described above. For the total HMPV-B dataset, the BMCMC chain was run for 500 million steps, with sampling every 1000 steps. The visualization of BSPs was presented by Tracer. The mean molecular evolutionary rates (substitutions/site/year) were also estimated using Tracer for the total dataset and for each sublineage separately. Statistical comparisons between sublineages were conducted using unpaired *t*-tests in EZR [27], with *p* < 0.05 considered statistically significant. The detailed parameters of the BSP analyses are shown in Supplemental Tables S3.

To identify sublineage-specific amino acid substitutions associated with the demographic changes, we analyzed all available sequences in our dataset for the B1 (*n* = 19) and B2 (*n* = 29) sublineages from the period of 2004–2009. This timeframe was selected to represent the phase following the major demographic fluctuations, based on the BSP results. Additionally, to evaluate the persistence of identified amino acid variations in recent years, single representative strains detected in 2022 for the B1 sublineage (GenBank accession no. PQ634879.1) and in 2024 for the B2 sublineage (GenBank accession no. PV052149.1) were included in the analysis. All sequences were aligned and visually compared with the B1 and B2 prototype strains (GenBank accession nos. EU857572.1 and EU857574.1, respectively).

### 2.4. Phylogenetic Distance Analysis

We assessed the genetic diversity of the HMPV-B *F* gene by calculating patristic distances. To this end, maximum likelihood (ML) phylogenetic trees were reconstructed independently for three distinct datasets: (i) all 500 HMPV-B strains combined, (ii) strains within the B1 sublineage, and (iii) strains within the B2 sublineage. Each tree was inferred using IQ-TREE version 2.2.2.6 [28], which utilized ModelFinder for automatic model selection and assessed nodal support via 1000 ultrafast bootstrap replicates and the SH-aLRT. Subsequently, all pairwise patristic distances, which represent the number of nucleotide substitutions per site, were calculated from each respective tree using Patristic [29]. The differences in mean distances between the groups were statistically evaluated with an unpaired *t*-test in EZR, where *p* < 0.05 was set as the threshold for significance.

### 2.5. Analysis of Codon-Specific Selective Pressures

We investigated codon-specific selective pressures on the HMPV-B F protein by analyzing the 500 *F* gene coding sequences via the Datamonkey web server (http://www.datamonkey.org/, accessed on 4 July 2025) [23]. The ratio of non-synonymous (*d*N) to synonymous (*d*S) substitution rates per site (*d*N/*d*S) was estimated to infer selective pressures. Evidence for codons evolving under positive selection (*d*N/*d*S > 1) and negative (purifying) selection (*d*N/*d*S < 1) was investigated using four complementary methods: Single-Likelihood Ancestor Counting (SLAC), Fixed-Effects Likelihood (FEL), Internal Fixed-Effects Likelihood (IFEL), and Fast Unconstrained Bayesian Approximation (FUBAR). For the likelihood-based methods (SLAC, FEL, and IFEL), a *p*-value of <0.05 was considered statistically significant. For the Bayesian method (FUBAR), a posterior probability of >0.9 was used as the threshold for significance.

### 2.6. Amino Acid Identity Analysis

We assessed the sequence identity of HMPV-B F proteins against an HMPV reference using a sliding-window approach, conceptually consistent with previous molecular evolutionary analyses [30,31]. This analysis represents smoothed identity values averaged across an 80-amino-acid window. First, we generated a 50% consensus sequence for each HMPV-B sublineage. Pairwise amino acid identity was then calculated between these consensus sequences and the HMPV reference (HMPV-A, GenBank: NC_039199.1) using a window size of 80 residues with a step size of 5 residues. From the resulting smoothed identity curves, we specifically calculated the mean percent identity for the core regions of neutralizing antibody binding sites. For the purpose of this study, these core regions were defined based on previous reports [17,32,33,34,35,36,37] as follows: site Ø (amino acids 54–65 and 170–183), site I (amino acids 23–31 and 282–284), site II (amino acids 233–238), site III (amino acids 277–281), site IV (amino acids 395–405), and site V (amino acids 136–156). This analysis was implemented in Python version 3.12.11 within the Google Colaboratory environment, utilizing the pandas library version 2.2.2 for data manipulation [38] and matplotlib version 3.10.0 for plotting [39]. Furthermore, site-specific amino acid mutation frequencies at neutralizing antibody binding sites (sites Ø and I–V) were calculated using Python for all 500 HMPV-B F protein sequences. Mutation frequencies were computed separately for the B1 and B2 sublineages and compared across sites.

### 2.7. Structure Modeling

Three-dimensional (3D) structural models of the HMPV F protein were generated for representative strains of B1 (GenBank: EU857572.1, PQ634879.1) and B2 (GenBank: EU857574.1, PV052149.1) using a local installation of ColabFold (LocalColabFold version 1.5.3) [40]. These strains were selected to encompass both the historical and contemporary genetic diversity within each sublineage. Specifically, for both B1 and B2, the oldest available sequence in the dataset was designated as the ancestral representative (EU857572.1 and EU857574.1), while the most recently sampled strain belonging to the most recently diverged subclusters in the time-scaled phylogeny was selected to represent contemporary viral evolution (PQ634879.1 and PV052149.1). For each prediction, a multiple sequence alignment (MSA) was first created locally against the uniref30, PDB100, and colabfold_envdb sequence databases. Subsequently, structural models were predicted by utilizing information from known protein structures as templates. To improve physical realism, the resulting structures underwent a refinement process using the AMBER force field. From the five models generated for each protein, we selected the one with the most favorable combination of the predicted local distance difference test (pLDDT), template modeling (TM)-score, and root mean square deviation (RMSD) metrics. The selected structures were visualized using PyMOL version 3.0.3 (https://www.pymol.org/).

### 2.8. B-Cell Epitope Prediction and Comparative Analysis

We predicted conformational B-cell epitopes on the F protein models of representative strains using four methods: DiscoTope 3.0 (higher confidence: 1.50, recall up to ~30%) [41], ElliPro (cutoff: 0.5) [42], SEPPA 3.0 (cutoff: 0.089) [43], and SEMA (cutoff: 0.76) [44]. An amino acid residue was defined as a putative B-cell epitope if it was predicted by at least three of the four methods. The identified epitopes were then mapped onto the 3D protein models using PyMOL. To complement the 3D structural analysis, we performed a multiple sequence alignment of the representative strains. Additionally, we visualized amino acid substitutions associated with predicted epitopes and experimentally identified neutralizing antibody binding sites in a linear sequence format. The reference strain for the linear sequence was the oldest strain in the dataset (B2 strain detected in 1982; GenBank accession: EU857574.1).

## 3. Results

### 3.1. Time-Scaled Phylogeny and Divergence Time Estimation

**Figure 1:**
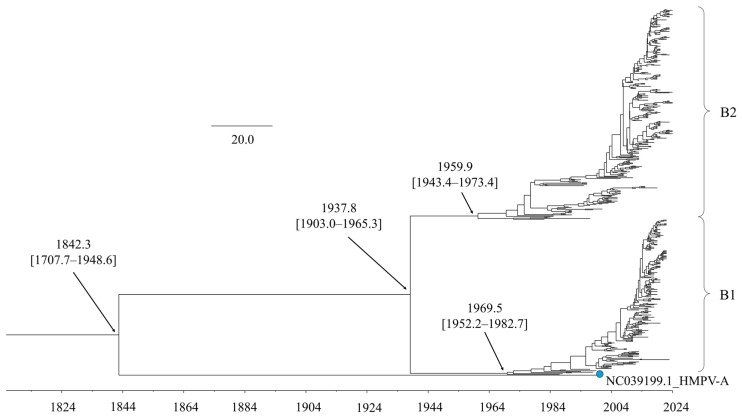
Time-scaled phylogenetic tree of the HMPV-B *F* gene inferred using the BMCMC method. The scale bar indicates time (in years). Numbers in parentheses denote the 95% HPD interval.

The time-scaled phylogenetic tree, constructed using the BMCMC method, revealed the evolutionary history of the HMPV-B *F* gene (Figure 1). The tree indicated that HMPV-A and HMPV-B diverged from a common ancestor around 1842 (95% Highest Posterior Density [HPD] interval: 1707.7–1948.6). Within HMPV-B, the divergence into the two primary sublineages, B1 and B2, was dated to around 1937 (95% HPD: 1903.0–1965.3). Sublineage B1 subsequently diversified into multiple subclusters starting around 1969. Similarly, sublineage B2 began diversifying into its own distinct subclusters around 1959.

### 3.2. Historical Changes in Effective Population Size

**Figure 2:**
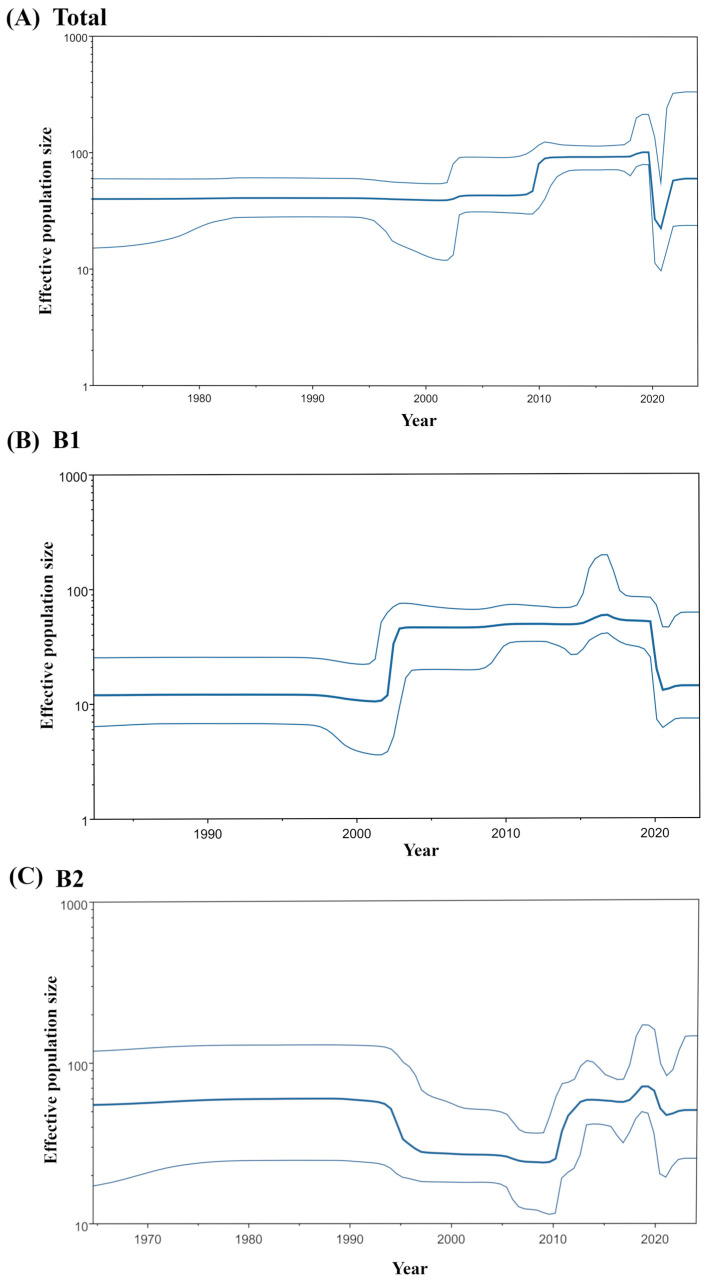
BSP analyses of the HMPV-B *F* gene. The plots show the phylodynamics for (**A**) all HMPV-B strains, (**B**) the B1 sublineage, and (**C**) the B2 sublineage. The y-axis represents the effective population size, and the x-axis represents time in years. The solid blue line indicates the mean posterior value, while the thin blue lines represent the 95% Highest Posterior Density (HPD) intervals.

A BSP analysis of the HMPV-B *F* gene revealed temporal dynamics in the virus’s effective population size (Figure 2). The overall effective population size of HMPV-B grew rapidly circa 2010 and then stabilized until 2019, when it sharply declined. A subsequent resurgence was observed around 2021, but it did not reach the previous peak (Figure 2A). The two sublineages displayed distinct demographic histories. The B1 sublineage showed a pronounced U-shaped pattern, characterized by a sharp rise around 2002, followed by a steep decline around 2020 that reduced its effective population size to a level comparable to that of the early 2000s (Figure 2B). Conversely, the effective population size of the B2 sublineage experienced a sharp decline around 1995, remained relatively stable, and then expanded sharply again around 2010. After a period of further growth in the late 2010s, the B2 population also decreased around 2020; however, this decline was less pronounced than that observed in B1, leaving the population size at a level similar to that of the early 2010s (Figure 2C).

To investigate whether sublineage-specific amino acid substitutions contributed to the contrasting population dynamics of the B1 (post-expansion) and B2 (post-contraction), we analyzed sequences from 2004 to 2009, a period during which the effective population sizes of both groups remained relatively stable. We compared these strains to representative early strains (B1: 1987; B2: 1984) and to recent strains collected from 2020 onwards (B1: 2022; B2: 2024). In the B1 sublineage, two amino acid substitutions (D475E and R479K) were identified in strains from the 2004–2009 period (Supplementary Figure S2). These substitutions persisted in representative strains from 2020 onwards, a period characterized by a sharp decline in effective population size. Similarly, the B2 sublineage exhibited one substitution (K143T) common to strains from 2004–2009 (Supplementary Figure S3). This mutation was maintained in representative strains from 2010 onwards, coinciding with the period of rapid population expansion.

### 3.3. Estimated Evolutionary Rate

Bayesian analysis estimated the molecular evolutionary rate of the *F* gene among all HMPV-B strains at 1.01  ×  10^−3^ substitutions/site/year (mean; 95% HPD, 8.73  ×  10^−4^ to 1.15 × 10^−3^). The evolutionary rate for the B1 sublineage (mean, 9.76 × 10^−4^; 95% HPD, 8.28 × 10^−4^ to 1.13  ×  10^−3^ substitutions/site/year) was significantly higher than that observed for the B2 sublineage (mean, 7.35 × 10^−4^; 95% HPD, 5.79  ×  10^−4^ to 8.93  ×  10^−4^ substitutions/site/year) (*p* < 0.001).

### 3.4. Phylogenetic Distance

**Figure 3:**
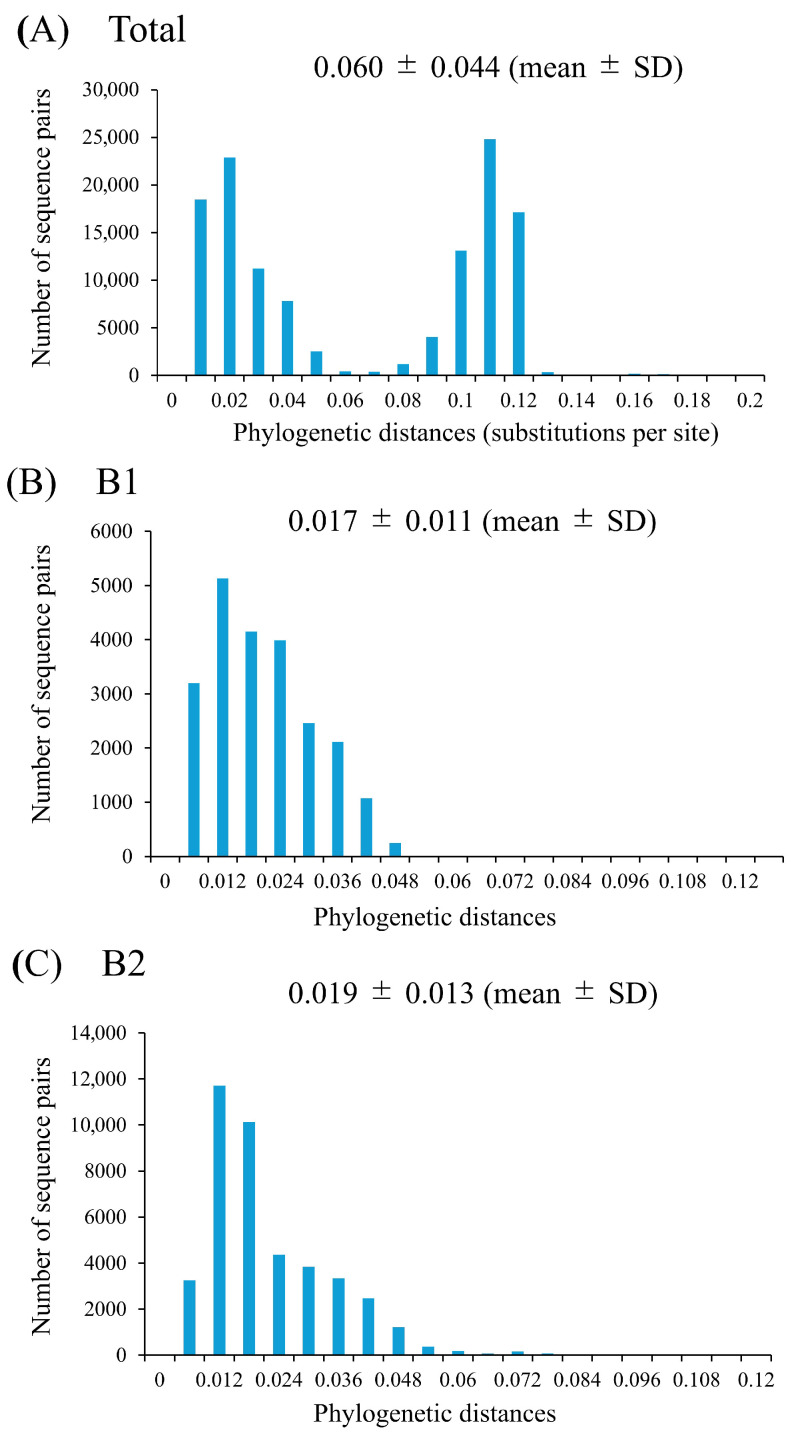
Phylogenetic distances for the HMPV-B *F* gene of all strains (**A**), B1 (**B**), and B2 (**C**). The *y*- and *x*-axes indicate the number of sequence pairs and phylogenetic distances (substitutions per site), respectively.

Pairwise patristic distances were calculated from the ML phylogenetic tree to assess the genetic diversity among the analyzed strains. The distribution of these distances for the entire HMPV-B dataset was bimodal, indicating clear divergence between sublineages, with a mean genetic distance of 0.060 (standard deviation [SD], 0.044) (Figure 3). When analyzed separately, the mean intra-sublineage distance was 0.017 (SD, 0.011) for B1 sublineage and 0.019 (SD, 0.013) for B2 sublineage. A statistical comparison confirmed that the genetic diversity within B2 was significantly greater than that within B1 (unpaired *t*-test, *p* < 0.0001).

### 3.5. Positive and Negative Selection Sites

The selective pressures acting on the HMPV-B F protein were investigated by analyzing its coding sequence for sites under positive and negative selection. While three methods (SLAC, IFEL, and FUBAR) each identified a single, unique codon under positive selection (codons 522, 143, and 482, respectively), no site was consistently identified by more than one method. This lack of consensus suggests limited evidence for strong positive selection acting on any specific site in the F protein. In contrast, strong evidence for purifying (negative) selection was observed. A total of 209 codons, corresponding to approximately 39% of the protein, were consistently identified as being under negative selection by all four methods. These negatively selected sites were distributed throughout the F protein sequence (Supplementary Figure S4).

### 3.6. F Protein Sequence Identity

**Figure 4:**
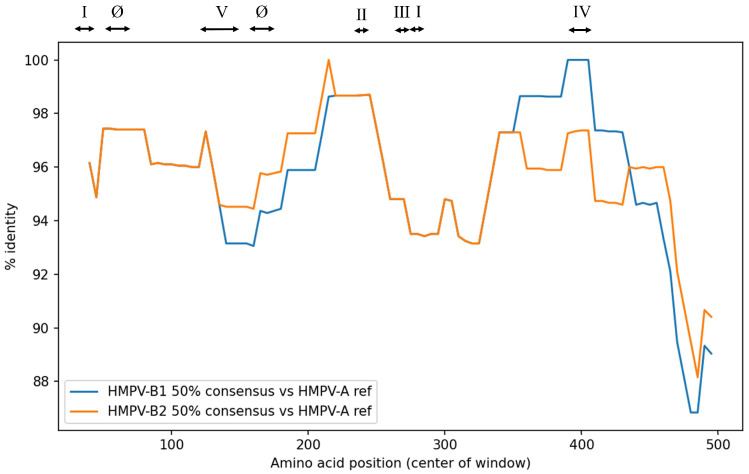
Sequence identity between HMPV-B F proteins and the HMPV reference strain. The plot compares the sequence identity of HMPV-B sublineages B1 (blue) and B2 (orange) against the HMPV reference strain. The y-axis represents the percent sequence identity, and the x-axis represents the amino acid position. Roman numerals (Ø, I–V) at the top indicate the approximate locations of neutralizing antibody binding sites.

The sequence identity of the F protein in the analyzed HMPV-B strains was compared to the HMPV reference strain (HMPV-A). The sliding-window identity profiles of HMPV-B1 and B2 showed only minimal differences across large portions of the F protein, particularly in the N-terminal region and mid-protein segments, resulting in partially overlapping curves (Figure 4). Consistent with this, the average sequence identity was high and nearly identical between the two sublineages (B1: 95.6% vs. B2: 95.7%) (Supplementary Table S4). High homology with HMPV-A reference strain was also maintained across the neutralizing antibody binding sites (sites Ø and I–V), ranging from 94.6% to 98.2%, with B1 and B2 exhibiting comparable identity values. The greatest difference between sublineages was observed at site IV, where the B1 sublineage showed 98.2% identity compared with 96.0% for the B2 sublineage.

At the residue level, we analyzed site-specific amino acid variation frequencies within neutralizing antibody binding sites (sites Ø and I–V) and compared dominant residues between the B1 and B2 sublineages (Supplementary Table S5). The dominant residues across these sites were largely shared between the two groups. Within each sublineage, amino acid variation at these sites was generally rare, being observed in only a small number of strains. Notable sublineage-specific differences were observed at residue 179 (site Ø), residue 280 (site III), residue 396 (site IV), and residue 143 (site V), where the predominant residues differed between B1 and B2 (B1: R179, D280, R396, and Q143; B2: K179, N280, Q396, and T143). At these positions, the B2 sublineage showed relatively higher within-sublineage heterogeneity; in particular, approximately 20% of B2 sequences carried the corresponding B1 residues at sites III and IV (D280 and R396, respectively).

### 3.7. Structural Modeling and Conformational Epitope Mapping

**Figure 5:**
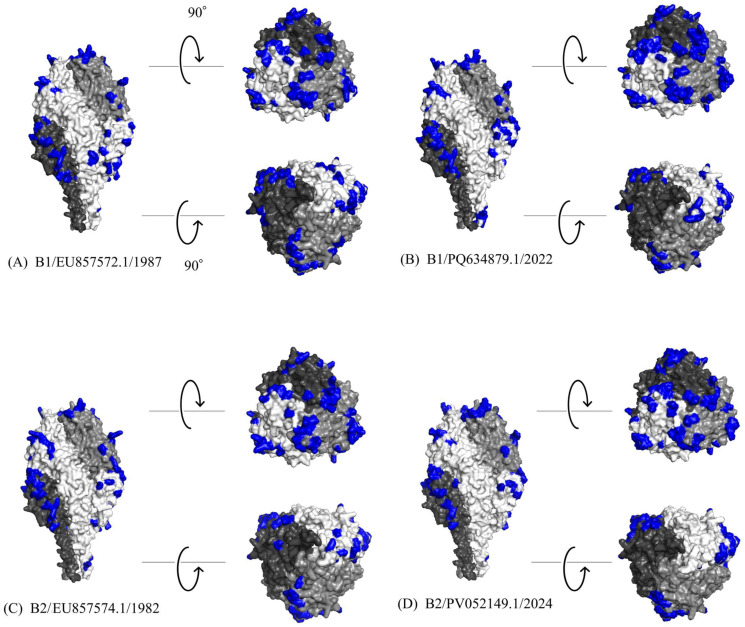
Structural models of representative HMPV-B F proteins. Each panel displays the predicted structure of a specific F protein: (**A**) a B1 sublineage strain from 1987 (EU857572.1), (**B**) a B1 sublineage strain from 2022 (PQ634879.1), (**C**) a B2 sublineage strain from 1982 (EU857574.1), and (**D**) a B2 sublineage strain from 2024 (PV052149.1). In each model, the three chains of the trimeric structure are colored light gray, dim gray, and black. Predicted conformational B-cell epitopes are highlighted in blue.

**Figure 6:**
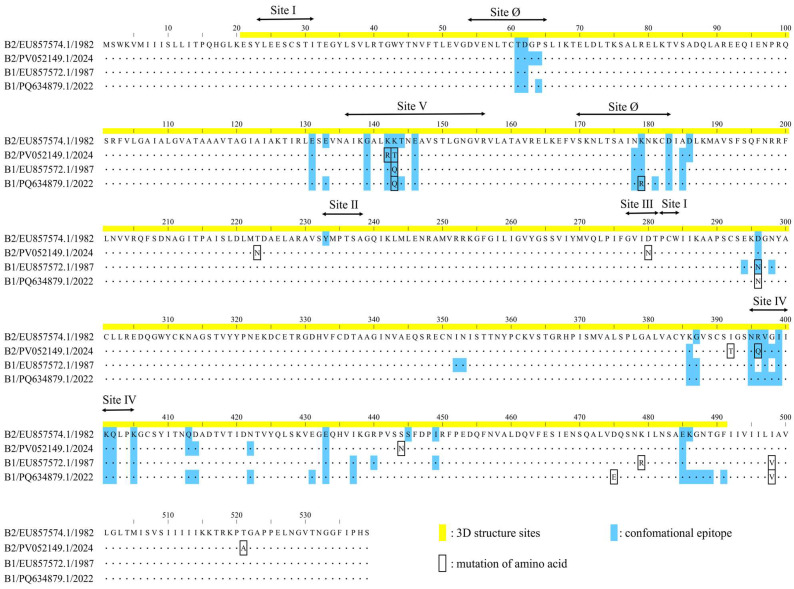
Amino acid sequence alignment of representative HMPV-B F proteins. The sequence region corresponding to the 3D structural model is highlighted in yellow. Amino acid substitutions among the aligned strains are enclosed in black boxes. Predicted conformational B-cell epitope sites are highlighted in light blue.

To investigate the antigenic properties of the F protein, 3D models were generated for representative HMPV-B strains, and predicted conformational B-cell epitopes were mapped onto their surfaces (Figure 5A–D). The predicted trimeric structures consistently adopted a “tree-like” conformation, comprising a globular head and a stalk region. The calculated conformational epitopes were predominantly located on the globular head region across all analyzed strains. A high degree of conservation was observed in both the amino acid sequences and the predicted epitope regions between the B1 and B2 sublineages. While the predicted models showed a high degree of structural similarity, specific amino acid substitutions were identified within calculated epitope sites (Figure 6). Notably, these included substitutions at experimentally identified neutralizing antibody binding sites: K142R and K143T in the 2024 B2 strain (PV052149.1); K143Q in both the 1987 B1 strain (EU857572.1) and the 2022 B1 strain (PQ634879.1); K179R in the 2022 B1 strain; and R396Q in the 2024 B2 strain. Despite these substitutions, the overall surface of the predicted epitopes did not appear to be substantially altered. A comprehensive list of the predicted epitope sites is provided in Supplementary Table S6.

## 4. Discussion

This study aimed to elucidate the evolutionary trajectory of the HMPV-B *F* gene and to assess potential alterations in antigenic sites associated with the accumulation of genetic mutations. Our phylogenetic analysis revealed that HMPV-B diverged into the B1 and B2 sublineages around 1937, after which both groups continued to diversify into distinct subclusters. The intra-sublineage genetic diversity (measured by patristic distances) within both sublineages remained limited. Our selective pressure analysis demonstrated that the *F* gene was predominantly under purifying selection, with negative selection sites distributed throughout the protein. Despite this overall conservation at the amino acid level, several substitutions were identified, including some located within experimentally identified neutralizing antibody binding sites. However, these substitutions were not predicted to substantially alter the conformational epitope regions. These findings suggest a high degree of predicted antigenic conservation in the HMPV-B *F* gene under strong evolutionary constraint.

The BSP analysis revealed notable fluctuations in the effective population size of HMPV-B. The overall effective population size for the *F* gene exhibited a sharp expansion around 2010, a trend that coincides with increased global reporting of respiratory infections [45]. This expansion may therefore be correlated with enhanced diagnostic capabilities and surveillance efforts, rather than being solely attributable to intrinsic viral factors. In addition, the sharp decline in effective population size around 2019 is likely attributable to decreases in HMPV case detection during the SARS-CoV-2 pandemic. Interestingly, the two sublineages displayed divergent demographic histories. B1 largely followed the overall HMPV-B pattern, whereas B2 exhibited a more irregular history, characterized by an earlier reduction followed by a later expansion. To investigate whether any shared amino acid substitutions were associated with characteristic changes in effective population size, we examined residues that were altered in both sublineages. Although several shared substitutions appeared in strains from the stable period (2004–2009), these changes persisted across later demographic shifts, including the B1 decline around 2020 and the B2 expansion around 2010. Accordingly, no shared amino acid changes were uniquely linked to periods of population contraction or expansion.

The overall evolutionary rate of the *F* gene in HMPV-B was estimated at 1.01 × 10^−3^ substitutions/site/year. This rate is higher than previously reported estimates for the *F* gene across all HMPV genotypes (7.12 × 10^−4^) and also exceeds those of other Pneumoviridae family members, such as RSV-A (7.69 × 10^−4^) and RSV-B (7.14 × 10^−4^) [30,31,46]. Moreover, it also surpasses the evolutionary rates reported for the *F* genes of other RNA viruses, such as human parainfluenza virus type 1 (HPIV1; 8.50 × 10^−4^), HPIV2 (4.20 × 10^−4^), HPIV3 (9.40 × 10^−4^), HPIV4 (4.41 × 10^−4^), and mumps virus (5.0 × 10^−4^) [47,48,49,50,51]. In contrast, amino acid variation in the F protein remained limited, indicating that increases in nucleotide-level diversity were not reflected at the protein level. Selective pressure analysis supported this observation, revealing pervasive purifying selection and no consistent evidence of positive selection across methods. As a consequence of this predominance of purifying selection, most substitutions were synonymous, resulting in broad conservation of the HMPV-B F protein despite its relatively high evolutionary rate. Similarly, although the evolutionary rate of the HMPV-B1 *F* gene was significantly higher than that of HMPV-B2, this higher rate did not translate into greater amino acid diversity between the two sublineages.

HMPV and RSV cause clinically similar respiratory illnesses and affect overlapping patient populations. As with RSV, the development of vaccines and prophylactic antibody preparations for HMPV is advancing, with particular focus on targeting neutralizing antibody binding sites on the F protein (sites Ø and I–V). These antigenic sites are of special interest because several correspond to cross-reactive neutralizing antibody epitopes shared between HMPV and RSV [16,17,18,34]. Because amino acid substitutions can alter antigenicity, we examined whether substitutions occurring within these neutralizing antibody binding sites, as well as across strains of different sublineages and detection years, could modify conformational epitope structures using in silico analysis. Our analysis showed that the predicted conformational epitope regions were highly conserved across all representative HMPV-B strains. Although several amino acid substitutions were observed within neutralizing antibody binding sites (e.g., sites III, IV, and V), these changes were not predicted to substantially alter the three-dimensional epitope architecture. Additionally, sequence identity across all 500 strains showed uniformly high amino acid conservation, with no major differences between the B1 and B2 sublineages. These results indicate that amino acid variation across the broader dataset was limited, consistent with the findings obtained from representative strains. These observations support the feasibility of developing vaccines and prophylactic antibody preparations targeting the F protein. Moreover, because most individuals are infected with HMPV by five years of age [1], the high structural conservation of these epitopes suggests that recurrent epidemics are more likely driven by waning host immunity than by viral antigenic drift. Consequently, preventive strategies targeting the conserved F protein are likely to remain effective in the long term. In contrast, the high degree of conservation does not necessarily confer an advantage for vaccine or antibody development. For instance, residues Asn57 and Asn172 at site Ø form the core of the glycan shield [19], and no mutations were detected at these positions in our representative strains. The preservation of this glycan shield suggests that developing antibody preparations targeting the apical region—an approach effective for RSV—may remain challenging for HMPV.

Although our consensus analysis did not identify strong evidence for positive selection, the IFEL method individually flagged codon 143 as a potential site. To complement the selection analysis, we examined site-specific residue frequencies within neutralizing antibody binding sites across the full dataset. In this frequency-based analysis, residue 143 in site V showed a clear sublineage-specific shift in the dominant residue (B2 ancestral strain detected in 1982: K143; B1: Q143; B2: T143). Importantly, sublineage-specific dominant residues were not confined to site V; they were also observed in regions corresponding to major neutralizing antibody binding sites, including sites III and IV (e.g., residue 280 in site III and residue 396 in site IV). With the exception of site III, which was not predicted as a conformational epitope by our criteria, the predicted epitope surfaces encompassing these regions appeared broadly conserved across representative structural models. Nevertheless, it is important to distinguish predicted structural conservation from functional consequences [52]. A single substitution could alter antigenicity by affecting the binding affinity of neutralizing antibodies, even if the overall three-dimensional structure is maintained. Moreover, our site-specific frequency analysis also identified a small subset of strains carrying substitutions within neutralizing antibody binding sites, and such minority variants could expand under changing immunological pressures, potentially affecting the performance of antibody-based interventions [20]. Therefore, continued molecular surveillance of HMPV remains warranted.

This study has several limitations. First, the use of publicly available sequences introduces potential sampling bias, as regions or periods with stronger surveillance are more likely to be represented. Second, our consensus-based epitope analysis did not identify site III, a known neutralizing antibody binding site comparable in importance to site IV. To enhance reliability, we defined residues as epitopes only when at least three of four prediction algorithms supported them. Although this stringent criterion reduces false-positive predictions, it likely resulted in the omission of certain antigenic sites, including site III. Finally, the present study lacks experimental validation. While epitope prediction and structural modeling provide useful insights, determining the quantitative functional impact of amino acid substitutions ultimately requires in vitro assays such as neutralization and membrane fusion studies, which remain important areas for future investigation.

## 5. Conclusions

Our analyses show that the HMPV-B *F* gene evolves under strong purifying selection, resulting in limited amino acid diversity between the B1 and B2 sublineages. Neutralizing antibody binding regions remained highly conserved, and substitutions within these sites were not predicted to alter epitope architecture. These findings indicate that the antigenic structure of the F protein is stable across decades, supporting its suitability as a target for antibody-based interventions.

## Data Availability

The original contributions presented in this study are included in the article/Supplementary Material. Further inquiries can be directed to the corresponding authors.

## Supplementary Materials

The following supporting information can be downloaded at: https://www.mdpi.com/article/10.3390/microorganisms14020396/s1. Figure S1: Temporal distribution of HMPV-B strains by collection year; Figure S2: Amino acid sequence alignment of representative F proteins from the HMPV-B1 sublineage; Figure S3: Sequence alignment of representative HMPV-B2 strains; Figure S4: Negative selection sites in HMPV-B F protein; Table S1: HMPV-B strains randomly excluded due to dataset downsizing; Table S2: List of HMPV-B strains and associated metadata used in this study; Table S3: Parameter settings for the BMCMC analyses; Table S4: Pairwise sequence identity of HMPV-B F proteins compared to the HMPV-A reference strain; Table S5: Site-specific amino acid variation frequencies and dominant residues within neutralizing antibody binding sites; Table S6: Predicted conformational B-cell epitopes on the HMPV-B F protein.
