# Supplementary material for: Molecular Evolution of the Fusion (F) Genes in Human Metapneumovirus Genotype B
Source: Microorganisms. 2026 Feb 6;14(2):396. doi: 10.3390/microorganisms14020396 (PMC12942750; doi:10.3390/microorganisms14020396)
Supplement: Supplementary file 1 [file microorganisms-14-00396-s001.zip › Supplementary_figure_20251222.pdf]

Supplementary data

Molecular Evolution of the *Fusion* (*F*) Genes in Human Metapneumovirus Genotype B

Tatsuya Shirai[1, 2, 3], Fuminori Mizukoshi[4], Mitsuru Sada[2], Kazuya Shirato[1], Takeshi Saraya[2], Haruyuki Ishii[2], Ryusuke Kimura[5], Toshiyuki Sugai[6], Akihide Ryo[4*], and Hirokazu Kimura[3, 7*]

[1] Department of Respiratory Viruses, National Institute of Infectious Diseases, Japan Institute for Health Security, Musashimurayama-shi 208-0011, Tokyo, Japan; shirai.t@jihs.go.jp (T.Shi.); shirato.k@jihs.go.jp (K.S.)

[2] Department of Respiratory Medicine, Kyorin University Faculty of Medicine, Mitaka-shi 181-8611, Tokyo, Japan; rainbow_orch@ks.kyorin-u.ac.jp (M.S.); saraya@ks.kyorin-u.ac.jp (T.Sa.); h141@ks.kyorin-u.ac.jp (H.I.)

[3] Advanced Medical Science Research Center, Gunma Paz University, Takasaki-shi 370-0006, Gunma, Japan; h-kimura@paz.ac.jp (H.K.)

[4] Department of Bioinformatics and Integrative Omics, National Institute of Infectious Diseases, Japan Institute for Health Security, Musashimurayama-shi 208-0011, Tokyo, Japan; mizukoshi.f@jihs.go.jp (F.M.); ryo.a@jihs.go.jp (A.R.)

[5] Gunma Prefectural Institute of Public Health and Environmental Sciences, Maebashi-shi, Gunma 371-0052, Gunma, Japan; kimura-r56@pref.gunma.lg.jp (R.K.)

[6] Department of Health and Welfare, Faculty of Health and Welfare, Prefectural University of Hiroshima,
Mihara-shi, Hiroshima 723-0053, Japan; sugai@pu-hiroshima.ac.jp (T.Su.)

[7] Department of Health Science, Gunma Paz University Graduate School of Health Sciences, Takasaki-shi 370-0006, Gunma, Japan

* Correspondence: ryo.a@jihs.go.jp; Tel.: +81-42-561-0771 (A.R.); h-kimura@paz.ac.jp; Tel. +81-27-365-3366 (H.K.)

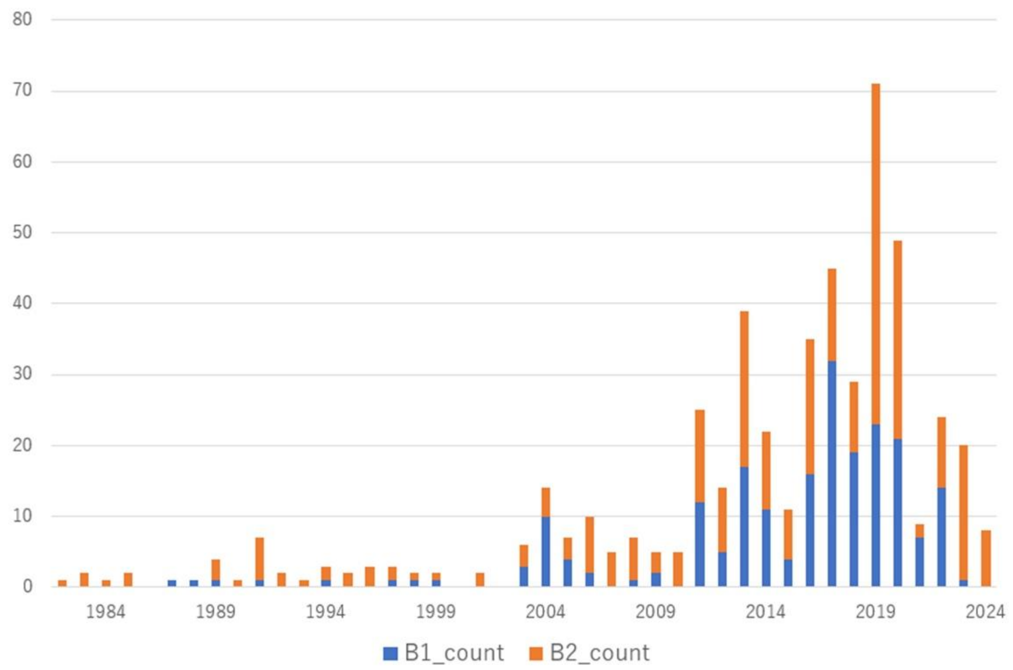

Figure S1. Temporal distribution of HMPV-B strains by collection year. The x-axis represents the collection year, and the y-axis indicates the number of strains. Bars representing the B1 and B2 sublineages are shown in blue and orange, respectively.

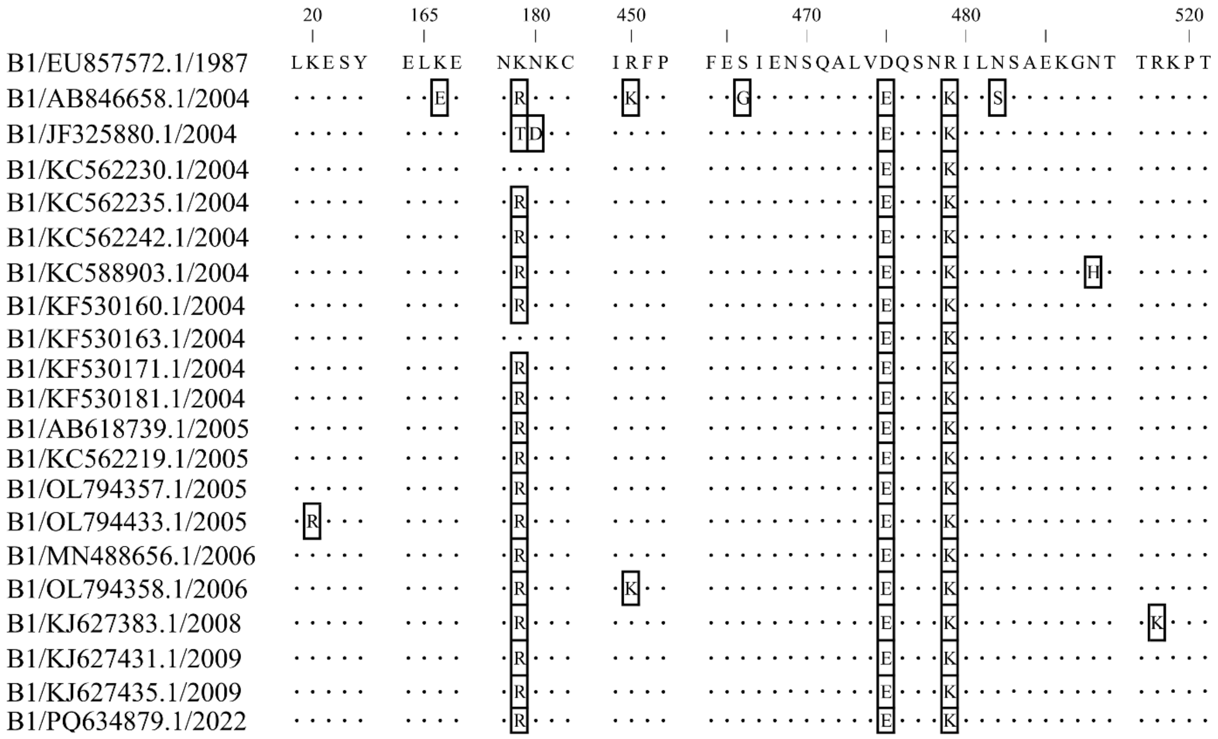

Figure S2. Amino acid sequence alignment of representative F proteins from the HMPV-B1 sublineage. Amino acid substitutions identified among the aligned strains are enclosed in black boxes.

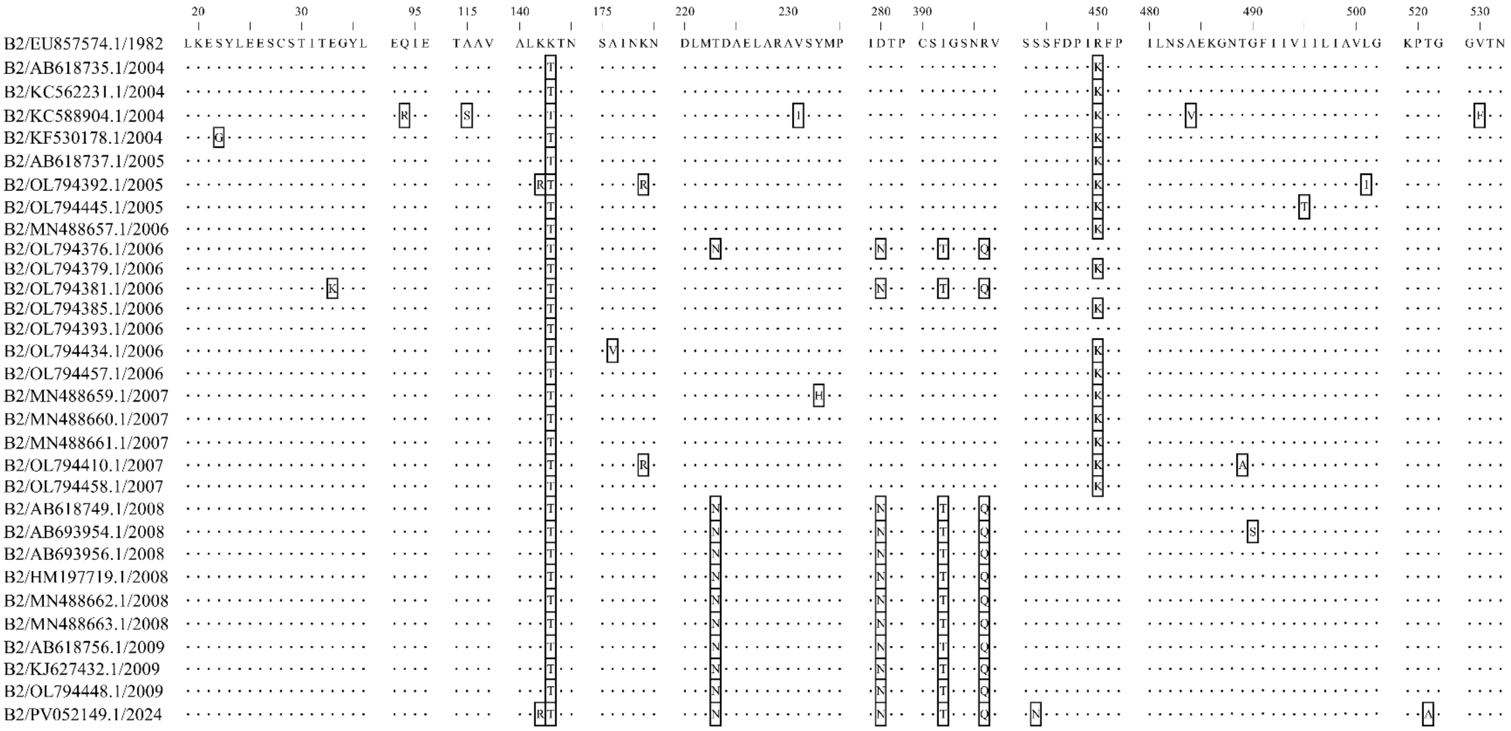

Figure S3. Sequence alignment of representative HMPV-B2 strains. Amino acid substitutions relative to the ancestral strain are highlighted with black boxes.

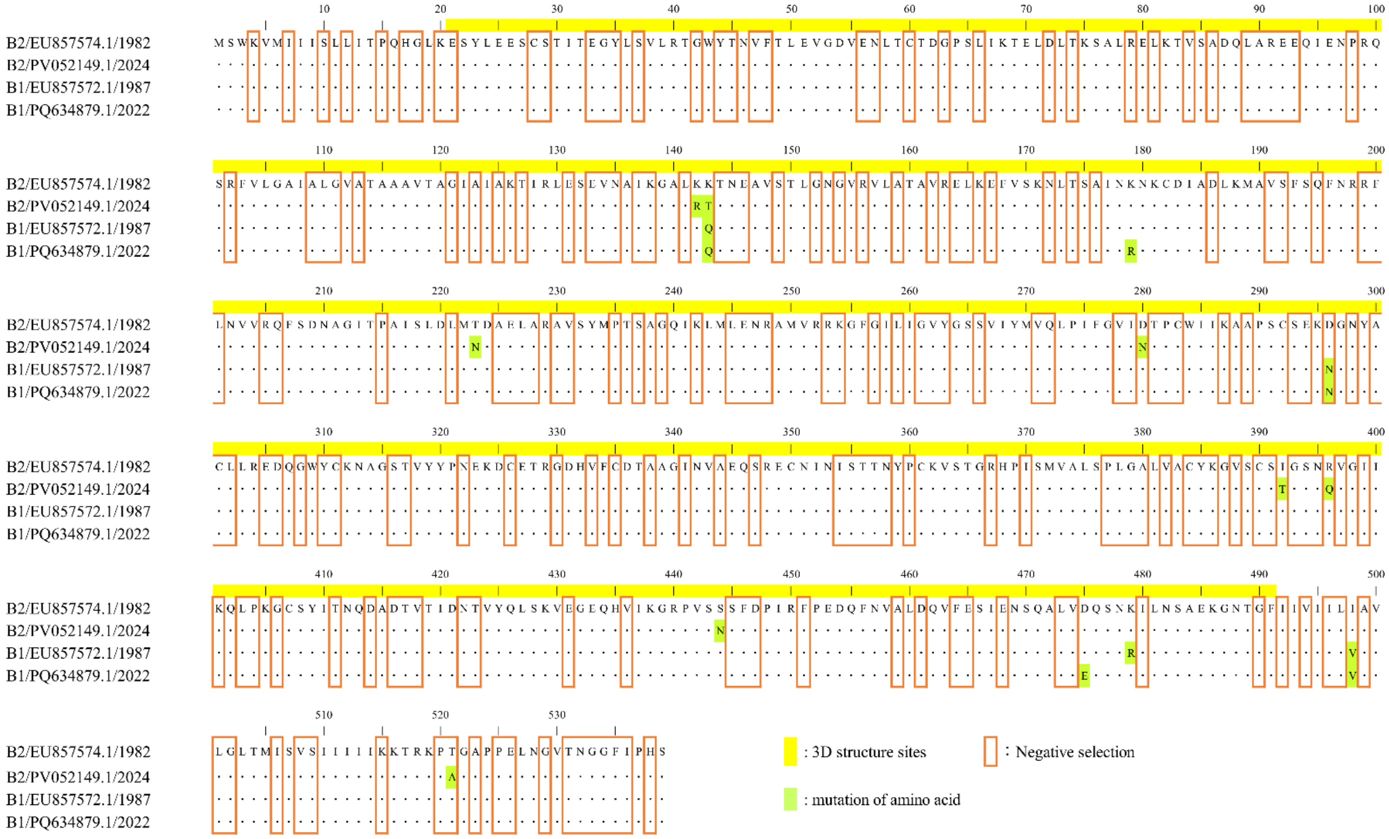

Figure S4. Negative selection sites in HMPV-B F protein. The sequence region corresponding to the 3D structural model is highlighted in yellow. Sites identified as being under negative selection are enclosed in light brown boxes. Amino acid substitution sites among the aligned strains are highlighted in light green.
